# Pain in amaXhosa women living with HIV/AIDS: a cross-sectional study of ambulant outpatients

**DOI:** 10.1186/s12905-017-0388-9

**Published:** 2017-04-13

**Authors:** Romy Parker, Jennifer Jelsma, Dan J Stein

**Affiliations:** 1 Department of Health & Rehabilitation Sciences, Faculty of Health Sciences, University of Cape Town, Anzio Road, Observatory, 7925 Cape Town, South Africa; 2 Department of Psychiatry & Mental Health, MRC Unit on Anxiety & Stress Disorders, Groote Schuur Hospital, Faculty of Health Sciences, University of Cape Town, Cape Town, South Africa

**Keywords:** Prevalence, Pain, HIV/AIDS

## Abstract

**Background:**

Pain is one of the most commonly reported symptoms in people living with HIV/AIDS, whether or not they are receiving anti-retroviral therapy. A recent systematic review identified a paucity of studies exploring pain in women in low and middle income countries. The prevalence and characteristics of pain in women living with HIV/AIDS may differ from that of men as many chronic pain conditions are more prevalent in women. The aims of this study were to establish pain prevalence, characteristics and management in amaXhosa women living with HIV/AIDS. In addition, we aimed to identify whether there were associations between pain in this population and the psychosocial factors of employment, education, self-efficacy, depression, post-traumatic stress disorder, health related quality of life and childhood trauma.

**Methods:**

A cross-sectional study of 229 women who had undergone HIV testing and were registered patients at a community health centre was conducted. Data were collected by interview with a demographic questionnaire, the Brief Pain Inventory-Xhosa, Childhood Trauma Questionnaire–Xhosa, Harvard Trauma Questionnaire–Xhosa for PTSD, Self-Efficacy for Managing Chronic Disease 6-Item Scale-Xhosa; the EQ-5D health related quality of life instrument, and the Beck Depression Inventory.

**Results:**

170 of the women had pain, a prevalence rate of 74.24% (95%CI 68.2 – 79.47%). The women reported significant pain with pain severity of 5.06 ± 1.57 and pain interference of 6.39 ± 1.96 out of 10. Only two women were receiving adequate pain management according to the pain management index. Participants reported a mean of 2.42 ± 1.21 different anatomical sites of pain. There were more unemployed participants in the group with pain and they had significantly fewer years of schooling. Those with pain had lower self-efficacy; health related quality of life and increased depression and PTSD symptom severity.

**Conclusion:**

This study highlights that pain is a common problem for amaXhosa women living with HIV/AIDS. These data emphasise the need to prioritise pain assessment and management in amaXhosa women living with HIV/AIDS. Routinely assessing for the presence of pain in women with HIV/AIDS has the potential to improve pain management and minimise the impact of pain on function.

## Background

Pain is one of the most commonly reported symptoms in people living with HIV/AIDS (PLWHA), whether or not they are receiving highly active anti-retroviral therapy (HAART) [1]. In a recent systematic review, we reported the pooled one-week prevalence of pain in PLWHA as 60.81% [95%CI (58.55–63.025)] with several studies reporting on the inadequate management of pain in this population [1–6]. In the systematic review we highlighted the high proportion of studies reporting on pain prevalence in ambulatory PLWHA from high income countries where men make up the majority of the infected population and transmission is predominantly through homosexual contact and intravenous drug use. The review identified a paucity of studies exploring pain in ambulatory PLWHA in low and middle income countries, and in particular in women in these countries. In addition to a lack of information on the prevalence of pain in women living with HIV/AIDS, previous studies lacked a systematic methodological approach indicating a need for studies using the STROBE guidelines [7].

The prevalence of pain in women living with HIV/AIDS may differ from that of men as many chronic pain conditions are more prevalent in women [8]. While these higher prevalence rates may be a reflection of cultural acceptance for women to report pain, it may be a consequence of differences in physiology between men and women. Physiological differences between men and women contribute to different responses to nociceptive input and women are known to have different responses to pharmacological and non-pharmacological treatments for pain [9–11]. Therefore, not only may the prevalence of pain in women living with HIV/AIDs differ from that in men, the characteristics of that pain may also be different.

In addition to pain being different between men and women, the prevalence, characteristics and impact of pain may differ in different cultural groups. Several studies have reported on the effects of culture and gender on pain experience in different ethnic and social groups [12, 13]. Studies exploring differences between African-American and Caucasian American groups have identified differences in pain prevalence in both healthy adults and chronic pain populations with African-American populations having a higher prevalence of chronic pain [12, 14]. Further, differences in patterns of pain prevalence in PLWHA from different cultures have been recorded in studies in South African populations [15]. In addition to differences in pain prevalence in different cultures, prevalence of pain in people living with HIV/AIDS has also been associated with depression, a history of trauma and low levels of quality of life [2, 5, 16], and a greater number of symptoms are associated with a lack of income [17]. All these risk factors are widespread in South African society.

In South Africa, not only is the HIV+ population predominantly female (60%), the majority have also not completed secondary level education, both factors which may well increase their risk for pain [5]. If the influence of gender and ethnic group on pain prevalence is acknowledged, then pain research which focusses on cultural and ethnic gender-specific groups is apt. Previous studies conducted exploring the prevalence of pain in PLWHA in South Africa have considered people from the Zulu, Venda and Tswana groups cultures [5]. The Xhosa are the largest cultural group in the Western Cape of South Africa with a population of approximately nine million people, approximately two million who have HIV. Members of the Xhosa refer to themselves as amaXhosa while the language is referred to as isiXhosa.

The lack of evidence on pain in women living with HIV/AIDS and particularly in women from developing countries, led us to design a study with the aim of establishing pain prevalence, characteristics (sites and severity) and management in amaXhosa women living with HIV/AIDS. In addition, we aimed to identify whether there were associations between pain in amaXhosa women living with HIV/AIDS and the psychosocial factors of employment, education, self-efficacy, depression, health related quality of life and history of trauma (PTSD or childhood trauma).

## Method

A clinical analytical descriptive study was conducted using a cross-sectional design.

### Setting and Subjects

This study was conducted in the community of Khayelitsha, South Africa. At the time of this study, this community had an estimated population of half a million people of whom 75% were under the age of 35 [18]. Overall, the unemployment rate as estimated to be 60–70%. The majority of residents of this community lived in shacks made from corrugated iron while 30% lived in houses built on separate plots. Nearly 40% of residents did not have water at their homes and made use of communal taps and communal toilets. Up to 75% of residents had electricity with the remainder relying on paraffin stoves and lanterns for cooking, heat and light [18].

The community health centre (CHC) where the study was conducted falls under the provincial department of health. The centre provides emergency services, has a midwife and obstetric unit, a pharmacy, an HIV HAART clinic, physiotherapy, occupational therapy and a school health service among others. The HAART clinic at this community health centre is well established with over 4000 patients attending each month for free monitoring and treatment [19]. The study focused on amaXhosa women from this community, living with HIV/AIDs and receiving treatment at the CHC.

Following identification of a CHC with an established HAART clinic as a site for data collection, sample size was calculated based on the lowest expected prevalence of pain. Between 1 July 2009 and 31 July 2009, 2356 women were treated at the HIV clinic. Using the 60.81% prevalence reported in the systematic review as the lowest expected prevalence of pain, sample size was calculated using Epi Info® (Version 7). For a population of 2356, using a 5% precision and a confidence interval of 90%, a sample of 234 was required to ensure adequate power. Based on these data, a target of 250 participants was set for the study.

A sample of convenience was utilized. AmaXhosa women aged 18 to 40 years, who had undergone HIV testing and were registered patients at the CHC HAART clinic, were approached to take part in a study exploring quality of life in amaXhosa women living with HIV/AIDS. Inclusion criteria comprised being female, a first language IsiXhosa speaker, HIV positive (all clinical stages) and between 18–40 years old. Women over the age of 40y were excluded from the study as the prevalence of co-morbidities such as osteoarthritis, hypertension and diabetes increase from this age which would potentially confound the results [20]. As information was gathered by interview, literacy was not a requirement. Exclusion criteria included having moderate to severe intellectual disability as recorded in their medical records, or cognitive impairment restricting ability to participate in an interview.

A research assistant (RA) fluent in IsiXhosa and English was employed to conduct the data collection by interview. All interviews were conducted in IsiXhosa.

### Procedure

Ethical approval was obtained from both the Faculty of Health Sciences Research Ethics Committee of the University of Cape Town (HREC Ref: 420 2007) and the Province of the Western Cape Department of Health (Reference number: 19/18/RP12/2009).

Recruitment of participants took place during clinic hours between Mondays and Fridays from 8 am to 2 pm. These are the peak times of the clinic and recruitment was aimed at all those attending the clinic for scheduled appointments. Women attending for routine monitoring of their condition were approached by the RA while waiting for their files to be processed. The RA invited the women to take part in a study exploring quality of life in PLWHA which would involve a one-hour interview prior to their routine clinic consultation. Pain was expressly not mentioned in the recruitment process. Patients who expressed an interest were provided with an information sheet and were given the opportunity to have any questions answered by the RA prior to completing informed consent.

Participants were interviewed by the RA in a private setting. Data were collected using a demographic questionnaire to establish age, level of education, employment, marital status and health status including time since diagnosis, CD4+ count and HAART management as well as the presence of co-morbidities including opportunistic infections. The Brief Pain Inventory-Xhosa (BPI-Xhosa) [21], the Childhood Trauma Questionnaire–Xhosa (CTQ-Xhosa), The Harvard Trauma Questionnaire–Xhosa (HTQ-Xhosa), the Self-Efficacy for Managing Chronic Disease 6-Item Scale (SE-6-Xhosa); the EQ-5D health related quality of life instrument, and the Beck Depression Inventory (BDI) were administered by interview. The BPI-Xhosa is a useful instrument for measuring the prevalence of pain as its first question asks: “Throughout our lives, most of us have had pain from time to time (such as minor headaches, sprains, and toothaches). Have you had pain other than these everyday kinds of pain during the last week?” The BPI-Xhosa is a valid measure of pain in urban amaXhosa women with good internal reliability [21]. The remaining instruments have also been validated for use in urban amaXhosa demonstrating good validity and reliability [22, 23]. The RA accessed the patient’s folder once all clinical documentation had been completed to obtain the most recent medical information including records of co-morbidities and/or opportunistic infections (diabetes, hypertension, peripheral neuropathy, tuberculosis, candidiasis, other) in the past year.

### Data analysis

The STROBE guidelines were used to inform the design, analysis and presentation of data [7]. Descriptive statistics were calculated using summary statistics and frequency distributions where appropriate. The prevalence of pain is reported as the percentage with 95% confidence intervals. The pain management index (PMI), a crude measure of the adequacy of pharmacological pain management, was calculated using the formula described by the developers of the BPI [24]. To calculate the PMI, PSS are assigned a score of 0, 1, 2, or 3 with PSS of 1–4 = 1, PSS of 5–6 = 2 and PSS of 7–10 = 3. Using the World Health Organisation guidelines, analgesic medications are allocated scores according to strength with no pain medication = 0, non-opioids = 1, “weak’ opioids = 2, and “strong” opioids = 3. The PMI score is calculated by subtracting the pain score from the analgesic score. A negative PMI score was considered an indicator of potentially inadequate pain management by the prescriber.

Data were examined for normality and subsequently comparisons were performed between those with pain (pain group) and those without pain (pain free group) for each of the numerical variables theorised to contribute to the presence of pain (self-efficacy, HRQoL, depression, childhood trauma and PTSD). Where data were normally distributed, differences between the groups (pain group and pain free group) were calculated using t-tests for parametric interval data and chi-squared tests for nominal and ordinal data; for data not normally distributed, differences between groups were calculated using the Mann–Whitney *U* test. The relationships between pain severity and numeric psychosocial variables were assessed using Spearman’s rank correlations. Spearman’s rho (r_s_) correlations were interpreted as weak (−0.3–-0.1 and 0.1–0.3), moderate (−0.5–-0.3 and 0.3–0.5) and strong (−1.0–-0.5 and 0.5–1.0) [25]. All data are presented as the mean ± standard deviation. Statistical significance was accepted as *p* < 0.05.

## Results

Data were collected between 1 February 2010 and 3 December 2010. No data collection took place between 19 August, 2010 and 16 Sept, 2010 due to a strike in the public health sector which disrupted service delivery at the research site. Data collection in the time immediately following the strike action was reduced due to lack of capacity at the clinic. Data collection was not conducted during the summer holiday month of December 2010 due to markedly decreased attendance at the clinic during this time (this decrease in turnover occurs on a yearly basis as a consequence of summer vacations).

### Socio-economic, demographic and clinical characteristics of the sample (*N* = 229)

**Fig. 1:**
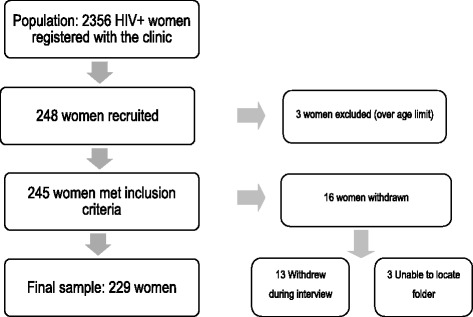
The sampling process

Of the 2356 women registered with the clinic, 248 were approached and recruited for the study, 245 met the inclusion criteria (three of those recruited were over the age limit) (Fig. 1). Data from 16 women were not included in the final analysis due to withdrawal during the interview or the RA being unable to trace the medical folder. The final sample consisted of 229 amaXhosa women with HIV/AIDS aged between 18–40 years of age. The mean age of the participants was 30.7 ± 4.8y with no difference in age between those with pain and those without pain. The majority of participants were single (61.57%) and could speak more than two languages.

### Prevalence of pain

In this sample of amaXhosa women living with HIV/AIDS, prevalence of pain was 74% (95%CI 68–79%) when the women were asked the first screening question from the Brief Pain Inventory–Xhosa.

### Characteristics of Pain

The full Brief Pain Inventory–Xhosa was completed by the 170 women who responded “Yes” to the screening question on the BPI-Xhosa. The Pain Severity Score (PSS) was calculated as the mean of the worst pain, least pain, average pain and pain now as described by the authors of the instrument [24]. Similarly, the Pain Interference Score (PIS) was calculated as the mean of the seven items relating to pain interference. As presented in Table 1, the 170 women with pain were experiencing moderate pain severity and moderate pain interference with function with severe episodes of pain (worst pain = 7.52 ± 1.73 and pain interference with enjoyment of life = 7.07 ± 2.46) [21].

**Table 1:** Pain Severity Scores and Pain Interference Scores from the BPI-Xhosa (*n* = 170)

	Mean ± SD
Pain Severity Score (mean of worst, least, average, now)	5.06 ± 1.57
Worst pain	7.52 ± 1.73
Least pain	4.65 ± 1.73
Average pain	3.93 ± 1.86
Pain now	4.14 ± 2.69
Pain Interference Score (mean of pain interference with activity, mood, walking, normal work, relations, sleep and enjoyment of life)	6.39 ± 1.96
Pain interference with activity	6.52 ± 2.56
Pain interference with mood	6.63 ± 2.76
Pain interference with ability to walk	6.18 ± 2.77
Pain interference with ability to do normal work	6.11 ± 2.68
Pain interference with relations with other people	5.89 ± 2.96
Pain interference with sleep	6.56 ± 2.78
Pain interference with enjoyment of life	7.07 ± 2.46

Pain Severity Scores and Pain Interference Scores range from 0–10 with higher scores indicating greater pain severity or greater pain interference with function

Data previously published in Parker at al (2016) [21]

#### Sites of pain

**Fig. 2:**
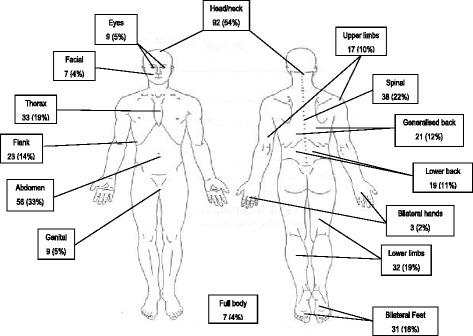
Frequency of most commonly reported painful anatomical areas. (Reprinted with permission, Copyright 1991 Charles S. Cleeland, PhD) [24]

On the body chart of the BPI, the women indicated a mean of 2.42 ± 1.21 (median 2; range 1–6) different anatomical sites of pain. The frequency of the most commonly reported different anatomical areas are presented in Fig. 2. The most frequently reported region of pain was the head/neck region (92 participants) followed by the abdomen (56 participants).

#### Pain management

**Fig. 3:**
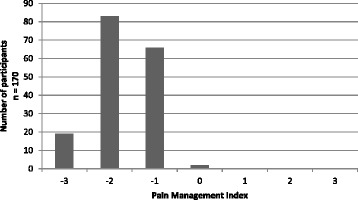
Pain Management Index Scores (*n* = 170)

The mean score on the Pain Management Index was −1.62 ± 0.66 (median −2, range −3–0). The distribution of PMI scores are presented in Fig. 3. Of the 170 women with pain, two were receiving adequate pharmacological analgesic management for their pain according to WHO guidelines.

The most commonly used analgesic was paracetamol, used by 104 (61%) of those with pain. Non-steroidal anti-inflammatory drugs (NSAIDs) were used by 10 (6%) while a combination of paracetamol and NSAIDs were used by 23 (14%) of those with pain. No analgesics were being used by 25 (15%) while the remainder were using a combination of paracetamol and adjuvant drugs (2), paracetamol and a weak opiate (5) and one woman was using paracetamol and a strong opiate. Pain relief obtained from medication is measured on the BPI on a 0%–100% VAS. The mean pain relief obtained from taking analgesic medication (*n* = 162) was 58.58 ± 18.87% (median 60%, range 0–100).

### Factors associated with pain

Those with pain had significantly less time at school than those without pain (10.18 ± 1.76 years of schooling vs. 10.93 ± 1.35 years of schooling; *t* = −2.98; *p* < 0.01). With regard to employment, 66% of the participants were unemployed with significantly more women with pain being unemployed (67% vs 62%; *χ* ^2^ = 16.99; *p* = 0.03). There was no difference between groups in health status (Table 2).

**Table 2:** Health status of participants with pain and without pain

	Pain group	Pain Free Group	
	Mean ± SD	Mean ± SD	Significance Test
Most recent CD4+ count	*n* = 16	*n* = 56	
cells/microL	5328.36 ± 206.97	338.68 ± 227.55	*t* = 0.31; *p* = 0.75
Time since diagnosis	*n* = 10	*n* = 40	
Years	4.3 ± 3.3	3.8 ± 3.2	*t* = 1.01; *p* = 0.31
Time since initiating treatment	*n* = 169	*n* = 57	
Months	24.5 ± 22.3	21.9 ± 18.1	*t* = 0.57; *p* = 0.57
	Number (%)	Number (%)	
Current Clinical Stage (I – IV)	*n* = 162	*n* = 55	*χ* ^2^ = 1.41; *p* = 0.23
HIV+ (Stage I and II)	59 (36)	25 (45)	
AIDS (Stage III and IV)	103 (64)	30 (55)	
Current HIV/AIDS Management	*n* = 169	*n* = 57	*χ* ^2^ = 12.12; *p* = 0.02
Monitoring	17 (10.00)	3 (5.08)	
First Line ARVs	137 (80.59)	44 (74.58)	
Second Line ARVs	12 (7.06)	3 (5.08)	
Pregnancy Prophylaxis	2 (1.18)	4 (6.78)	
Defaulted	1 (0.59)	3 (5.08)	
Opportunistic Infections			
Tuberculosis	19 (11)	2 (3)	*χ* ^2^ = 3.18; *p* = 0.07
Candidiasis	7 (4)	2 (3)	*χ* ^2^ = 0.06; *p* = 0.8
Other infections (genital herpes, papular pruritic eruptions, meningitis, pneumocystis pneumonia, arthralgia)	10 (6)	0 (0)	*χ* ^2^ = 3.63; *p* = 0.06

The psychosocial factors of self-efficacy, depression, health related quality of life, risk of PTSD or a history of childhood trauma which are known to contribute to the prevalence of pain were explored (Table 3). Compared to those without pain, the women with pain had worse scores for self-efficacy (*p* = 0.02), depression (*p* < 0.01); health related quality of life (*p* < 0.01) and risk of post-traumatic stress disorder (*p* = 0.03).

**Table 3:** Associations between psychosocial factors and pain (*N* = 229)

	Pain group (*n* = 170)	Pain Free Group (*n* = 59)	
	Mean ± SD	Mean ± SD	Significance Test
Self-efficacy	6.45 ± 1.60	7.00 ± .51	***t*** **= −2.4;** ***p*** = 0.02*
Health related quality of life (EQ5D)	0.683 ± 0.248	0.932 ± 0.101	***t*** **= −7.48;** ***p*** < 0.01*
Depression (BDI)	18.5 ± 10.4	12.6 ± 9.0	***t*** **= 3.9;** ***p*** < 0.01*
Harvard Trauma Questionnaire (PTSD)	1.78 ± 1.1	1.41 ± 1.1	***t*** **= 2.22;** ***p*** = 0.03*
Childhood Trauma	41 ± 9	39.8 ± 10	*t* = 0.85; *p* = 0.39

bold* indicates significance at *p* < 0.05

Following identification of differences in the numeric variables of number of years of schooling, self-efficacy, health related quality of life (EQ-5D index), depression (BDI) and PTSD in women reporting pain compared with those not reporting pain, relationships between each of these variables and pain severity (PSS) and pain interference (PIS) were explored using a correlation matrix (Table 4). There were weak to moderate negative relationships between both pain severity and interference, and years of schooling and health related quality of life (EQ5D index), and weak to moderate positive relationships between pain severity and interference and depression. There was a weak positive relationship between the women’s scores for PTSD and pain interference. Finally, there was no association between self-efficacy and with pain severity or interference.

**Table 4:** Correlation matrix for pain and biopsychosocial variables (*n* = 170)

	PSS	PIS	Years of school	SE-6-Xhosa	EQ5D index	BDI	HTQ
Pain Severity Score (PSS)	1.00						
Pain Interference Score (PIS)	0.35*	1.00					
Years of school	−0.25*	−0.20*	1.00				
Self-efficacy (SE-6-Xhosa)	−0.04	−0.13	0.04	1.00			
Health related quality of Life (EQ5D index)	−0.35*	−0.37*	0.25*	0.22*	1.00		
Depression (BDI)	0.20*	0.44*	−0.22*	−0.27*	−0.44*	1.00	
Post-traumatic stress disorder (HTQ)	0.10	0.19*	−0.12	0.11	−0.21*	0.36*	1.00

bold* indicates significance at *p* < 0.05

## Discussion

In this sample of amaXhosa women living with HIV, a pain prevalence rate of 74.24% (95%CI 68.2–79.47%) was recorded. The women were experiencing moderate pain with mean pain severity scores of 5.06 ± 1.57 and mean pain interference scores of 6.39 ± 1.96. The women reported a mean of 2.42 ± 1.21 different anatomical sites of pain on the body chart with the most common anatomical region of pain being the head/neck area (reported by 92 women) followed by abdominal pain (56 women). Pain severity and interference was correlated to years of education, health related quality of life and depression.

The 74% prevalence of pain in this sample was markedly higher than the 60% prevalence of pain reported in the systematic review of prevalence of pain in PLWHA [1]. However, this figure is similar to the 72% prevalence of pain reported in an ambulant HIV positive rural cohort of South Africans [5]. There are several factors which may explain why the pain prevalence rate was higher than the 60% obtained from the pooled data in the systematic review. The first lies with the gender of the sample. Several studies have identified differences in nociceptive pain processing between men and women in addition to higher prevalence of many different painful conditions in women [26–29]. Essentially, being female increases risk for developing pain. Thus the higher prevalence of pain in this sample may simply be a reflection of this increased risk for pain among women and an indication of how much being female increases risk for pain. The second factor relates to the socio-economic status of the women. Socio-economic status has also been identified as a risk factor for pain [30]. More than half of the women were unemployed (66%) and the significantly higher rates of unemployment in the women with pain suggest that this may have been a contributing factor. Finally, previous studies have identified low levels of education as increasing risk for pain [31, 32]. The majority of the sample in the present study had not completed all 12 years of schooling in South Africa, with those reporting pain having significantly lower levels of education than those without pain.

Previous studies have repeatedly found few links between disease parameters and pain in PLWHA [33]. This was also the case for the women in this study with no differences in clinical stage between those with pain and without pain and no differences in most recent CD4+ count and length of time since initiating treatment. There was a difference between groups in HIV/AIDS management with a larger proportion of those with pain only being monitored for their condition. However, given that there was no difference in CD4+ counts between the groups, it does not seem logical to suggest that an increase in pain is a consequence of a lack of treatment as the disease indicators do not correspond.

### Pain severity and pain interference

The mean PSS (5.06 ± 1.57) indicates that the women had moderate pain, pain recognised to interfere with cognition and function suggesting that the pain experienced by this group was severe enough to affect their quality of life and require treatment [34]. The mean score for the category of worst pain was 7.52 ± 1.73, indicating severe pain and greater levels of suffering. Considering the severity of pain these women were experiencing, it is particularly concerning that they were not receiving adequate analgesia. It can be suggested that the lack of pharmacological treatment may well have contributed to the scores for pain interference (6.39 ± 1.96) which indicates moderate interference with life roles as a result of pain. In an era where treatment of PLWHA has shifted to minimising the effect of symptoms in order to maximise quality of life, the under-treatment of pain in these women and its impact on their function is concerning.

There was only a moderate correlation between PSS and PIS; suggesting that pain severity is not the only factor contributing to how these women cope with their pain. This relationship between pain severity and pain interference reinforces the findings of Mphahlele and colleagues who reported on the stoicism of their participants who, despite moderate to severe levels of pain, reported low to moderate levels of PIS [5]. This was particularly notable in their participants from a rural area, with the authors theorising that perhaps living conditions were such that participants could not afford to allow pain to interfere with their function as this would impact on basic survival. The lack of association between pain severity and disability is recognised in the literature relating to musculoskeletal pain and cancer pain with disability relating more to psychosocial factors such as catastrophic thinking and beliefs about pain than with the severity of pain [35]. Thus in a disadvantaged group of women, it may be proposed that pain interference is influenced by a multitude of variables such as cultural beliefs, education and socio-economic level and is not simply a direct consequence of pain severity.

#### Sites of pain

The number of different sites of pain reported by the women on the body chart (>2) is a phenomenon which appears throughout previous studies describing pain in PLWHA [1]. The widespread anatomical sites of pain are noteworthy as the literature suggests that pharmacological management of pain in PLWHA focuses on painful peripheral neuropathy which presents with bilateral foot pain followed by bilateral hand pain when severe [36–43]. However, the varied sites of pain suggest that these women are suffering from causes of pain other than peripheral neuropathy. Their pain may be directly related to HIV, or, it may arise from unrelated co-morbidities such as osteoarthritis or be idiopathic in nature.

Pain is a cortical construct or output of the brain proposed to be produced by a threat neuromatrix (previously viewed as a pain neuromatrix) [44]. If pain is approached from a standpoint of being a construct in response to threat, it may be hypothesised that PLWHA are more vulnerable to suffering from pain, not as a direct result of increased sources of nociception, but as a consequence of the individual being under threat. This may explain the high prevalence rates of pain in this population and the multiple sites of pain reported by PLWHA. Approaching pain management from an understanding of pain as an expression of threat may be a useful theory in developing treatment plans for PLWHA with pain; treatment plans which include both pharmacological approaches but also non-pharmacological interventions. The data from this study and others on pain in PLWHA indicate that further research into effective management approaches is needed in order to address the widespread pain experienced.

### Pain management

While it must be acknowledged that the PMI is a crude measure of the adequacy of pharmacological pain management, it is notable that only two women in this study were receiving adequate analgesic therapy. This is not a unique finding and it is disconcerting considering that the under-treatment of pain in PLWHA has been raised in the literature for nearly 20 years [45, 46]. However, the pharmacological treatment of pain in PLWHA has limited efficacy [33] and non-pharmacological interventions such as those used in other chronic pain conditions are now proposed for this population [47]. Given the pattern of pain recorded in this study, particularly the varying sites of pain which suggest both HIV and non-HIV related causes, non-pharmacological treatments which target the biopsychosocial nature of pain appear relevant. These non-pharmacological treatments target the multiple variables (disease and psychosocial) which may contribute to pain in PLWHA and include exercise [48, 49], education [50, 51] and cognitive behavioural strategies [52]. While these pain treatments alone appear to have small effects sizes, a combination approach of exercise and education based on a chronic pain management approach and aimed at patient empowerment holds promise [47, 53].

#### Relationship between pain and other variables

The majority of the participants (58%) were classified as having AIDS. The CD4+ counts (330.98 ± 211.89cells/microL) suggest that their health was relatively stable at the time of interview, however, the range of CD4+ counts reflect a more diverse state of health in the sample with the most recent counts ranging from 20–1040cells/microL. These figures suggest that some participants were acutely ill although only 38 participants (17%) were currently suffering from an opportunistic infection. Given the wide ranging sites of pain and the prevalence of head/neck and abdominal pain, it is likely that the participants were suffering from pain not related to their HIV disease or opportunistic infections. In countries where ART has been made available, the profile of HIV/AIDS has changed from that of a terminal illness to one of a chronic debilitating disease which has the potential to become terminal [54]. In South Africa, a similar transition can be expected with growing numbers of people living with HIV/AIDS living for longer periods of time and PLWHA needing management of widely prevalent conditions such as arthritis, hypertension and diabetes [20]. Treatment of interdependent conditions such as these will require patient-centred approaches which integrate both pharmacological and non-pharmacological treatments to enhance quality of life.

Level of education as represented by number of years of schooling was negatively correlated to both pain severity and pain interference scores. This association between level of education and pain in PLWHA has previously been identified by several authors [2, 33]. Low levels of education are commonly associated with low levels of health literacy, poor nutrition, poor general health and unemployment [55]; the combination of which will contribute to individual’s vulnerability to illness and perceived threat when they are ill. Low levels of education are relevant when the literature emphasising the need to educate persons living with chronic diseases in order to minimise symptoms, improve adherence and HRQoL is considered [56]. To maximise treatment efficacy, educational interventions need to be developed for the appropriate educational level and consider the need for education on a broad range of health literacy topics.

In a cross-sectional study such as this one is not possible to determine whether HRQoL is influenced by the presence of pain or whether a low HRQoL increases risk of pain. While it appears intuitive to suggest that the presence of pain would reduce HRQoL, the study design limits exploration of directionality. This limitation is not restricted to the HRQoL data but similarly applies to the directionality of relationships between pain and employment, education, self-efficacy, depression and history of trauma. The mean score for depression on the Beck Depression Inventory of 17 ± 10.39 was concerning as a score of >13 can be regarded as indicative of depressive symptoms. When scores for depression were explored by group, those with pain remained above the 13-point score (18.54 ± 10.42) while those without pain were below this score (12.59 ± 9.04) suggesting that those with pain were depressed [57, 58]. The links between pain and depression are well recognised in the broader pain literature and in studies exploring pain in PLWHA [16, 59, 60].

The study’s specific aim was to explore pain in AmaXhosa women living with HIV/AIDs. The sample studied was drawn from a single site in an urban resource poor setting, introducing a selection bias which may have influenced results. The data should thus be interpreted with consideration of this bias.

## Conclusion

This study reinforces the high prevalence, severity and under-management of pain previously reported in PLWHA. It also highlights that pain appears to be a common problem for amaXhosa women living with HIV/AIDS. These data emphasise the need to prioritise the assessment and management of pain and psychiatric comorbidities in amaXhosa women living with HIV/AIDS and draws attention to the possible role for non-pharmacological management approaches. Routinely assessing for the presence of pain in women with HIV/AIDS has the potential to improve pain management and minimise the impact of pain on function.

## Abbreviations

BDI: Beck depression inventory
BPI-Xhosa: Brief pain inventory - Xhosa
CD4+: Cluster of differentiation 4
CHC: Community health centre
CTQ-Xhosa: Childhood Trauma Questionnaire - Xhosa
HAART: Highly active antiretroviral treatment
HIV/AIDS: Human Immunodeficiency Virus/Autoimmune Deficiency Syndrome
HRQoL: Health related quality of life
HTQ-Xhosa: Harvard Trauma Questionnaire - Xhosa
NSAIDS: Non-steroidal anti-inflammatory drugs
PIS: Pain interference score
PLWHA: People living with HIV/AIDS
PMI: Pain management index
PSS: Pain severity score
PTSD: Post-traumatic stress disorder
RA: Research assistant
SE-6-Xhosa: Self-efficacy for managing chronic disease 6-item scale - Xhosa
STROBE: STrengthening the Reporting of OBservational studies in Epidemiology
